# Auditory variant of Charles Bonnet Syndrome: A review of two clinical cases

**DOI:** 10.1192/j.eurpsy.2025.2058

**Published:** 2025-08-26

**Authors:** J. Santilari Planas, J. Tortajada Valero, E. García González

**Affiliations:** 1 Institut Pere Mata; 2 CIBERSAM; 3 Institut d’Investigació Sanitaria Pere Virgili, Reus, Spain

## Abstract

**Introduction:**

Charles Bonnet Syndrome (CBS) is characterized by complex hallucinations in patients with sensory impairment. The World Health Organization in 2018 outlined the diagnostic criteria for CBS as follows: complex visual hallucinations; partial or complete vision loss; and absence of mental disorders (Dhooge Patty et al. AES 2022;7:12). Notably, auditory hallucinations are excluded from this definition, and recent reviews argue that CBS should not encompass sensory or auditory hallucinations (Rojas LC, Gurnani B. StatPearls 2024). Although their frequent association with psychotic disorders, a broad differential diagnosis is crucial given the diverse etiologies. Recently, there has been growing literature of cases of auditory hallucinations which were only explained as a CBS.

**Objectives:**

The work aims to explore and discuss the auditory variant of Charles Bonnet Syndrome (CBSa).

**Methods:**

Based on two clinical cases, we conducted a literature review on this topic using PubMed database.

**Results:**

We describe two patients with severe sensorineural hearing loss and auditory hallucinations. The first patient, a 95-year-old independent in daily activities, presented with sudden-onset external voices. The second, an 81-year-old institutionalized woman, exhibited musical auditory hallucinations that developed over several months. Both patients maintained insight into the unreality of their hallucinations.

Comprehensive evaluations ruled out other potential causes, including neurological, psychiatric, pharmacological, and toxic-metabolic origins. Given the symptoms and and the exclusion of alternative diagnoses, CBSa was determined as the underlying cause.

The course of CBSa is known to be variable; hallucinations may diminish or resolve spontaneously or when the hearing deficit is ameliorated. Therefore, management is directed toward addressing the primary cause. No standardized treatment exists; however, medications such as antipsychotics, antidepressants, or antiepileptics may alleviate symptoms (Perez PA et al. Open Neurol J 2017 Feb 28;11:11-14). In our cases, both patients were treated with risperidone at 1 mg/24h, resulting in good tolerance and complete resolution of symptoms.

**Conclusions:**

Despite the absence of definitive diagnostic criteria for CBS, it is predominantly associated with visual hallucinations without concomitant neurological or psychiatric pathology, excluding auditory manifestations. However, if patients experience hallucinations in non-visual sensory modalities while retaining insight into their unreality, they should not be excluded from a CBS diagnosis. Recent case studies support the existence of such variants.

This paper advocates for the refinement of CBS diagnostic criteria to encompass these additional manifestations. Expanding these criteria could enhance psychiatric epidemiology by addressing the current underestimation of CBS prevalence and improving the recognition and management of this condition.

**Disclosure of Interest:**

None Declared

